# Preoperative obliteration of choroidal arteries in the treatment of large hypervascular tumors in the lateral ventricle

**DOI:** 10.1186/s12883-021-02129-4

**Published:** 2021-03-12

**Authors:** Yukihiro Yamao, Kazumichi Yoshida, Akira Ishii, Masahiro Tanji, Masakazu Okawa, Yohei Mineharu, Takayuki Kikuchi, Yoshiki Arakawa, Hiroharu Kataoka, Yasushi Takagi, Susumu Miyamoto

**Affiliations:** 1 Department of Neurosurgery, Kyoto University Graduate School of Medicine, ShogoinKawahara-cho, Sakyo-ku, 606-8507 Kyoto, Japan; 2 Department of Neurosurgery, National Cerebral and Cardiovascular Center, Suita, Japan; 3 Department of Neurosurgery, Tokushima University, Tokushima, Japan

**Keywords:** Hypervascular tumor, Embolization, Feeder obliteration, Lateral ventricle, Choroidal artery

## Abstract

**Background:**

Removal of large hypervascular tumors in the lateral ventricle still poses a surgical challenge. These tumors are usually fed from choroidal arteries, and vascular control is typically performed late during the removal. We aimed to evaluate the clinical efficacy of our strategy for persistent preoperative obliteration of feeders from the choroidal arteries to manage large hypervascular tumors in the lateral ventricle.

**Methods:**

We retrospectively analyzed six patients with hypervascular tumors in the lateral ventricle. We first attempted to obstruct feeders using endovascular treatment, and, if unavailable, performed initial microsurgical occlusion through the temporal horn for the staged tumor removal.

**Results:**

In all patients, feeder obliteration was successfully performed; the anterior choroidal arteries were occluded by the endovascular treatment and microsurgical occlusion in one and five patients, respectively, while the lateral posterior choroidal arteries were occluded via endovascular treatment in four patients. No patients had permanent symptoms due to feeder obliteration, and tumor devascularization was achieved at the mean rate of 69.9%. During the tumor removal, the mean blood loss volume was 253 ml. No postoperative hemorrhage had occurred, and all patients scored ≤ 2 on the modified Rankin Scale at six months post-removal.

**Conclusions:**

Although further studies are warranted, persistent feeder obliteration of choroidal arteries could be an effective treatment strategy against large hypervascular tumors in the lateral ventricle.

## Background

The surgical treatment of large hypervascular tumors in the lateral ventricle, such as meningiomas, remains challenging for neurosurgeons. These tumors are often detected when they have grown to huge tumors, and  hence due to their large size, cortical damage is a significant risk factor during surgical intervention. These tumors are mainly supplied from the anterior (AChA) and/or posterior choroidal arteries (PChA) [1–5], and during surgical resection procedures, vascular control is typically performed late. A recent review [6] has indicated that the postoperative mortality was associated with hematomas in the surgical bed, which is probably attributed  to rich tumor vascularization. Therefore, in the surgical management of hypervascular tumors in the lateral ventricle, preoperative obliteration of feeders from the choroidal arteries can potentially reduce  the difficulty of surgical resection and prevent postoperative hemorrhage. Endovascular feeder embolization is preferable due to its minimal invasiveness [7, 8]; however, a previous case report has shown that microsurgical occlusion of feeders originating from the choroidal arteries in the inferior horn of the lateral ventricle might be an effective method [9].

In the present study, we aimed to evaluate the clinical efficacy of the strategy of managing large hypervascular tumors in the lateral ventricle surgically, through persistent preoperative obliteration of feeders from the choroidal arteries; we first attempted to obstruct feeders using endovascular treatment; if unavailable, performed initial microsurgical occlusion in the temporal horn for the staged tumor removal.

## Materials and Methods

### Patient selection

We analyzed the six consecutive patients with large hypervascular tumors in the lateral ventricle who had undergone surgical intervention at our institute between April 2014 and April 2019. The variables analyzed included demographic characteristics, such as age and sex, and clinical characteristics, including symptoms, imaging results, surgical approaches undertaken, the extent of tumor resection, the results of pathological examination of the tumors, and any complications related to the surgical procedures.

### Surgical procedures for feeder obliteration

The artery feeding the tumor was confirmed using preoperative digital subtraction angiography (DSA) in all patients. The main target vessels for obliteration, preceding the tumor removal, were the AChA and PChA, which were sufficiently distal from the approach used for the tumor removal. First, feeder obliteration was performed by endovascular treatment (transarterial embolization; TAE), due to its minimal invasiveness, after assessment of diagnostic images by specialists certified by the Japanese Society for Neuroendovascular Therapy. If TAE was not available, initial microsurgical obliteration was performed for the staged tumor removal.

### TAE of the AChA or PChA

Using a transfemoral approach, a 6F or 7F guiding catheter was placed into the internal carotid or vertebral artery. Intravenous heparin was intermittently administered throughout the procedure, and serial activated clotting time (ACT) measurements were taken throughout the procedure to maintain the ACT above 200 seconds. A microcatheter (Marathon; Medtronic, Minneapolis, MN, USA) was advanced to the target vessel. To avoid ischemic complications, in the case of feeders projecting from the AChA, the catheter tip was advanced beyond the angiographic plexal point [10] and a provocative test was performed using lidocaine and a barbiturate agent under the local anesthesia. In the case of feeders projecting from the PChA, the catheter tip was advanced just proximal to the tumor. Embolization was performed using 100–300 µm trisacryl gelatin microspheres (Embosphere; Merit Medical Systems, Inc. UT, USA) and/or a variety of detachable coils.

### Microsurgical occlusion of feeders from the AChA

Microsurgical occlusion of feeders from the AChA was performed in the temporal horn using the subtemporal approach when a patient’s dominant side was affected to avoid complications resulting in language impairment [11]. In this approach, the temporal horn was accessed from the basal surface of the temporal lobe via the occipitotemporal sulcus. In cases where the patient’s non-dominant side was affected, the temporal horn was accessed via the middle temporal sulcus using the temporal approach. Within the temporal horn, the AChA was identified using neuronavigation and indocyanine green video angiography; it was then coagulated and cut distal to its entrance point into the temporal lobe, leaving its cisternal branches intact.

In all patients, postoperative magnetic resonance imaging (MRI) was performed, and the rate of devascularization of the tumor (inferred from the rate of reduction in gadolinium-enhanced lesion labeling compared to preoperative MRI) was calculated using neuronavigation software (Vector Vision Compact, BRAINLAB, Heimstetten, Germany).

### Tumor resection and outcomes

Tumor resection was usually performed within one week after feeder obliteration, before further pial feeders development. Surgical approaches were decided on the basis of the tumor location, with the parietal or temporal approaches most commonly selected.

The extent of resection was estimated based on review of pre- and postoperative MRI data using the neuronavigation software. Based on postoperative imaging, gross total resection was defined as excision without visible residual tumor and a lack of residual tumor detected in postoperative imaging; subtotal resection was defined as a ≤10 % remaining tumor; partial resection included 25–90 % resection of the tumor [8]. Postoperative symptoms and modified Rankin Scale (mRS) scores assessed six months after tumor removal were also analyzed.

## Results

Six consecutive patients, two males and four females, were assessed, with a median age of 56.5 years (range: 40–71 years). All tumors were located in the lateral ventricle; five tumors occurred in the trigone, while one in the body of the lateral ventricle. The mean maximum tumor diameter and volume were 5.5 cm (range: 4.7–6.1 cm) and 48.3 cm^3^ (range: 27.8–84.7 cm^3^), respectively. Based on preoperative DSA, feeding arteries had originated only from the AChA in two patients, from the AChA and the lateral PChA (LPChA) in two patients, and from the AChA, LPChA, and other arteries (such as an anterior cerebral artery, middle cerebral artery, and/or lenticulostriate artery) in two patients.

Preoperative clinical symptoms included contralateral hemiparesis in three patients, homonymous hemianopsia in three patients, and cognitive dysfunction (i.e., word-finding difficulty, short-term memory disturbance, and/or dyscalculia) in three patients.

A summary of patients’ demographics is described in Table 1.

**Table 1:** Patient demographics

	Location	Size (cm)/ volume (cm^3^)	Preoperative symptoms	Pathology	Postoperative symptoms	Pre/Postoperative mRS
1	Lt trigone	5.4/38.1	Cognitive dysfunction	WHO grade I meningioma	Cognitive dysfunction, homonymous hemianopsia	1/2
2	Rt trigone	5.9/38.3	None	WHO grade II meningioma	None	0/0
3	Lt trigone	5.1/27.8	Homonymous hemianopsia	WHO grade I meningioma	Homonymous hemianopsia	1/1
4	Rt trigone	5.9/84.7	Hemiparesis, homonymous hemianopsia	WHO grade I meningioma	Homonymous hemianopsia	1/1
5	Lt body	4.7/36.2	Cognitive dysfunction, hemiparesis	WHO grade I meningioma	Homonymous hemianopsia, cognitive dysfunction, hemiparesis	2/2
6	Rt trigone	6.1/64.5	Cognitive dysfunction, homonymous hemianopsia, hemiparesis	WHO grade II solitary fibrous tumor	Homonymous hemianopsia	1/1

*Lt* Left, *Rt *Right, *mRS *Modified Rankin Scale, *WHO *World health organization

### Feeder obliteration

In two patients (Patients 2 and 3), the feeding arteries were obstructed in a single session with either TAE or microsurgical occlusion of the AChA, while in  four patients, feeder obliteration was performed in two or more sessions, using both TAE of the LPChA and microsurgical occlusion of the AChA. As for the target vessels, the AChA was occluded by TAE in one patient and by microsurgical occlusion in five patients; the LPChA was occluded by TAE in four patients. Other arteries were occluded by either TAE or microsurgical occlusion.

Based on postoperative MRI, the mean devascularization rate of the tumors was 69.9 % (range: 33.8–98.6 %). Postoperative MRI showed small infarction of the thalamus, which was in the territory of the LPChA, in four patients, and of the internal capsule, which was in the territory of the AChA, in one patient. Three patients (Patients 2, 3, and 4) showed no new neurological deficits, while the other three (Patients 1, 5, and 6) developed slight, transient contralateral hemiparesis due to thalamic infarction or brain edema. However, no patients had permanent neurological deficits due to feeder obliteration. A summary of feeder obliteration and surgical outcomes is shown in Tables 1 and 2.

**Table 2:** Summary of the treatment

	Feeder	AChA diameter (mm)	Target vessels for obliteration		DWI positive post feeder obliteration in the territory of AChA, LPChA	Symptoms due to feeder obliteration	Devascularization rate after feeder obliteration (%)	Approach of the tumor removal	Blood loss volume (mL)	Removal
			TAE	Microsurgery						
1	AChA, LPChA, MCA	0.9	LPhA, MCA	AChA	Yes (LPChA)	Transient hemiparesis	33.8	Parieto-occipital	360	Total
2	AChA	1.8	AChA	-	No	No	97.9	Parietal	100	Total
3	AChA	0.9	-	AChA	No	No	98.6	Parietal	82	Total
4	AChA, LPChA	0.9	LPChA	AChA	Yes (LPChA)	No	78.5	Temporo-occipital	70	Subtotal
5	AChA, LPChA MPChA, LSA ACA	0.9	LPChA, MPChA	AChA, LSA, ACA	Yes (LPChA)	Transient hemiparesis	55.2	1. Parietal 2. Parieto-occipital	1. 270 2. 600	Partial
6	AChA, LPChA	1.2	LPChA	AChA	Yes(AChA and LCPhA)	Transient hemiparesis	55.2	1. Parietal 2. Parieto-occipital	1. 320 2. 220	Total

*AChA* anterior choroidal artery, *ACA* anterior cerebral artery, *DWI* Diffusion weighted imaging, *LPChA* lateral posterior choroidal artery, *LSA* lenticulostriate artery, *MCA* middle cerebral artery, *MPChA* medial posterior choroidal artery, *TAE* transarterial embolization

### Tumor removal and surgical outcomes

The mean time interval from the final feeder obliteration procedure to the tumor removal was three days (range: 1–6 days). Tumors were removed via a trans-parietal approach (including the parieto-occipital approach) in five patients, and a trans-temporal approach (including the temporo-occipital approach) in one patient. In two patients (Patients 5 and 6), since the tumor itself remained firm, further parieto-occipital approach was required in a subsequent session. The mean blood loss volume during tumor removal was 253 mL (range: 70–600 mL). Four patients underwent gross total resections, one underwent subtotal resection, and one underwent partial resection.

After the tumor removal, contralateral hemiparesis improved in two patients (Patients 4 and 6), while homonymous hemianopia developed in two patients (Patient 1 and 5); in Patient 5, contralateral hemiparesis and cognitive dysfunction worsened. In all patients, mRS scores assessed six months post-surgery were ≤ 2.

Pathological evaluations revealed World Health Organization (WHO) grade I meningiomas in four patients, a grade II meningioma in one patient, and a grade II solitary fibrous tumor in one patient.

**Fig. 1:**
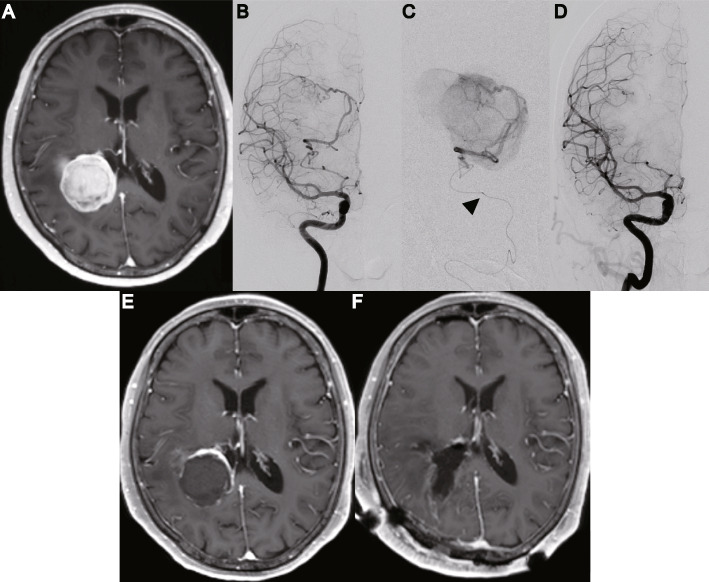
In Patient 2, a right lateral ventricular tumor was incidentally detected. **a **Preoperative magnetic resonance imaging (MRI) with gadolinium contrast showing a tumor in the right lateral ventricle (maximum diameter: 5.9 cm, tumor volume: 38.3 cm^3^). **b **Preoperative digital subtraction angiography (antero-posterior projection) reveals feeders originating solely from the anterior choroidal artery. The diameter of the AChA was 1.8 mm. **c **For endovascular treatment, a microcatheter is advanced beyond the angiographic plexal point (arrowhead). After a provocative test was performed using lidocaine and a barbiturate agent, the feeding arteries were obstructed using 100–300 µm trisacryl gelatin microspheres and coils. **d **Digital subtraction angiography (antero-posterior projection) reveals that the feeders projecting from the anterior choroidal artery have diminished after endovascular treatment. **e **After endovascular treatment, MRI shows a 97.9 % decrease of gadolinium-enhanced lesions. **f **After tumor removal via an intraparietal sulcus approach, MRI shows no residual tumor

**Fig. 2:**
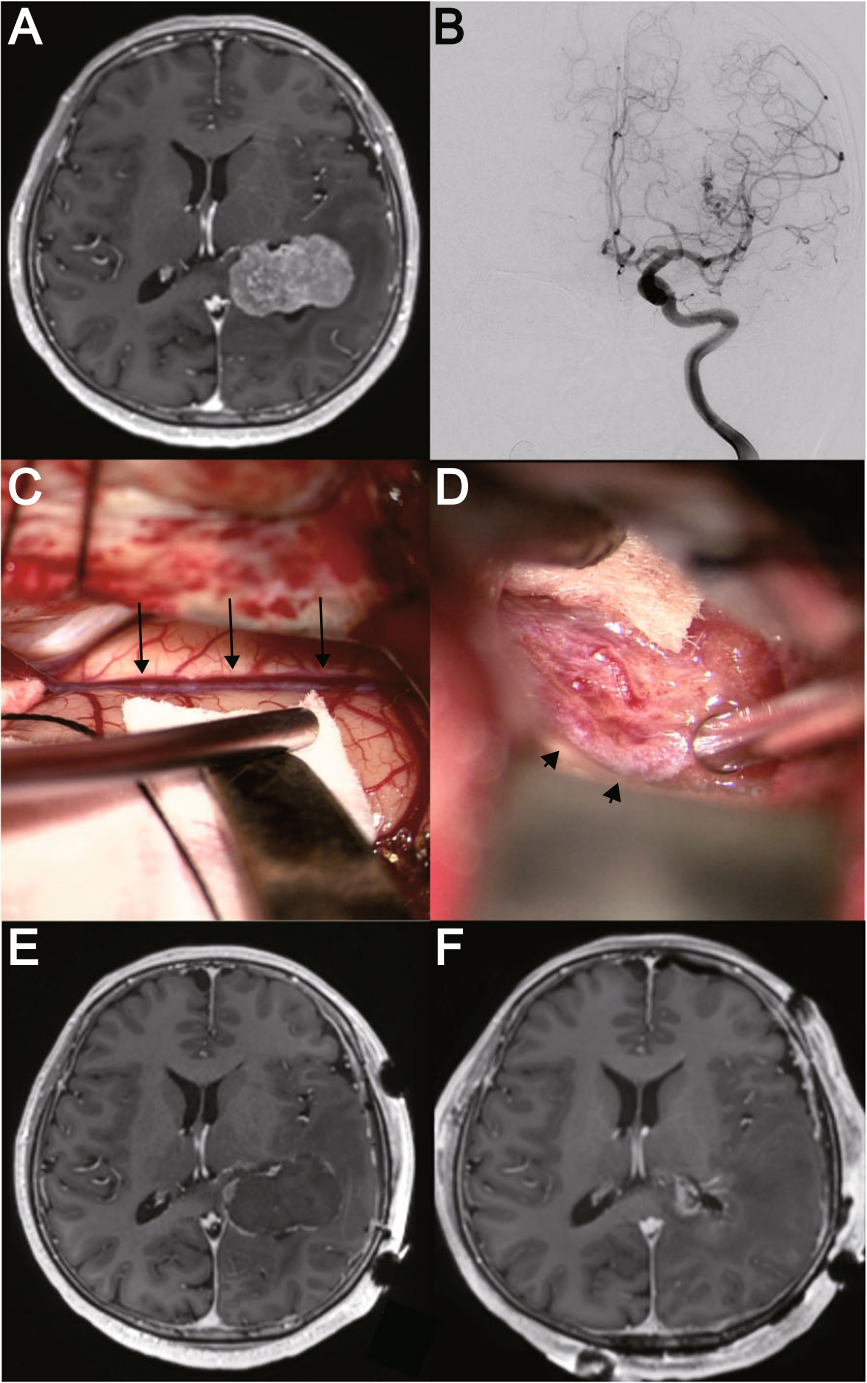
In Patient 3, a left lateral ventricular tumor was incidentally detected eight years prior. Since the patient was experiencing visual field impairment due to tumor growth, surgical intervention was planned. **a** Preoperative magnetic resonance imaging (MRI) scan with gadolinium contrast showing a tumor in the left lateral ventricle (maximum diameter: 5.1 cm, tumor volume: 27.8 cm^3^). **b** Preoperative digital subtraction angiography (antero-posterior projection) reveals feeders originating solely from the anterior choroidal artery. The diameter of the AChA was 0.9 mm, which was considered too small to cannulate. **c** After temporal craniotomy, the occipitotemporal sulcus (black arrows) has been identified. The temporal horn is accessed by a subtemporal approach. **d** In the temporal horn, the anterior choroidal artery has been identified (black arrowheads), and cut. **e** After microsurgical occlusion of the anterior choroidal artery, MRI shows a 98.6% decrease of gadolinium-enhanced lesions. **f** After tumor removal via a parietal (intraparietal sulcus) approach, MRI shows no residual tumor

A summary of treatment and surgical outcomes is shown in Tables 1 and 2. Illustrative cases are described in Figs. 1 and 2.

## Discussion

In this study, we presented a strategy for persistent feeder obliteration preceding the surgical removal of large hypervascular tumors in the lateral ventricle. Devascularization following feeder obliteration was achieved in all six patients, resulting in decreased blood loss during the subsequent tumor removal in the majority of the patients. Postoperative hemorrhage had not occured, and the outcomes were relatively favorable.

### Risks of obliterating choroidal arteries

The normal mean diameter of the AChA is 1.24 mm (range: 0.4–3.4 mm) [12]. The classic clinical symptoms caused by occlusion of the AChA are contralateral hemiparesis, hemisensory loss, and homonymous hemianopia [13]. The AChA is divided into the cisternal and plexal segments, and only the cisternal segment contains the perforating branches, which may be associated with the clinical symptomology [14]. The plexal point observed by DSA has been proposed to identify the entry point into the temporal horn; that is, the point separating the cisternal and plexal segments of the AChA.

Embolization of feeders arising from the AChA can carry the risk of ischemic complications [1, 2], and superselective embolization of choroidal arteries has been reported only in a few case reports of intraventricular meningiomas [15–17] and in a few studies of arteriovenous malformations [10, 18, 19]. When considering TAE, the catheter tip must be advanced beyond the plexal point to avoid ischemic complications; however, the risk of procedure-related ischemic or hemorrhagic complications is reportedly as high as 16.7–30.7 % [10, 18, 19]. In those studies, AChAs were dilated enough for deep catheterization, but the safety diameter of catheters was not identified. In our study, TAE was preformed successfully only in one patient in which the AChA’s diameter was 1.8 mm; in five other patients in whom the mean diameter of the AChA was 1.0 mm (range: 0.9–1.2 mm, Table 2), we instead selected microsurgical occlusion after assessing the diagnostic images. Thus, the safety diameter for deep cannulation of the AChA might be 1.5 mm.

The risk of ischemic complications might be avoided by performing microsurgical occlusion of the AChA in the temporal horn. Reported approaches for the temporal horn include the transsylvian, the temporal, and the subtemporal approaches [11, 20]. We mainly selected the subtemporal approach to reduce the risk of postoperative language dysfunction [11]. In the present series, no neurological permanent  deficits, including language dysfunction, were observed due to  microsurgical occlusion. In a cadaver study [21], it was shown that 38 % of the capsulothalamic artery arises from the first portion of the plexal segment. This anatomical variation could be associated with ischemic complications, even in cases involving the direct obliteration of the AChA. Actually, in our study, postoperative MRI in one patient (Patient 6) detected infarction in the internal capsule, suggesting that this anatomical variation could be associated with ischemic complications.

The LPChA branches to the choroid plexus and the trigone of the lateral ventricle. In addition, it also supplies the crus, commissure, body, and part of the anterior columns of the fornix, as well as the dorsomedial and pulvinar portion of the thalamic nucleus, and a part of the lateral geniculate body [22, 23]. Reports on patients with discrete LPChA infarction are limited, with a visual field defect, typically quadrantanopia or hemianopia, being the primary symptom [22]. Controlling ischemic complications post embolization of the LPChA may be challenging since no angiographic safety point has been identified thus far, unlike the AChA [10]. In our study, small thalamic infarction was detected in all four patients with endovascular feeder occlusion, although the catheter tip was advanced just proximal to the tumor in all patients. This was probably because either the blood flow was stolen towards the tumor and the small branches projecting to the thalamus were invisible, or the LPChA was retrogradely thrombosed. Further studies are warranted to identify the appropriate catheter position for avoiding these types of ischemic events.

### Role of feeder obliteration preceding tumor removal

Preoperative endovascular feeder embolization is commonly used for vascular-rich meningiomas, except for those located in the lateral ventricle, to reduce intraoperative blood loss in more devascularized tumors, and also to soften tumors [8, 24]. However, past studies have reported no significant differences in surgical duration, extent of resection, blood transfusion requirements, or measures of morbidity [8, 25]. In the present study, the mean devascularization rate was 69.9 %, and the mean blood loss volume (253 mL) during the tumor removal was much less than the volumes reported in a previous study of intraventricular meningiomas (mean: 530 mL; range: 100–1900 mL) in which feeder obliteration was not performed before the tumor removal [4].

The optimal time interval between feeder embolization and tumor removal remains controversial; it has been suggested that the removal should be performed approximately one week post embolization due to greater softening of the tumor, allowing for easier removal [26], whereas others have recommended within seven days post embolization to avoid the revascularization of tumors [27]. In this study, the mean time interval from the final feeder obliteration procedure to the tumor removal was three days (range: 1–6 days). In two patients (Patients 2 and 3) with extensively devascularized tumors (> 95 %), the tumors softened; in three patients (Patients 4, 5, and 6) with two or more feeder arteries, multiple small pial feeders were observed intraoperatively and the tumors were still very firm, even though all the main feeders had already been obstructed. The presence of small pial feeders may explain the low devascularization rates in tumors supplied by two or more feeders. In a previous case report, tumor volume decreased and the tumor softened 13 months post microsurgical occlusion of feeders arising from the AChA or PChA [9]. Therefore, if a patient’s neurological condition is not critical, it may be better to abstain from treatment until substantial tumor shrinkage is obtained following feeder obliteration in cases where tumors are supplied by multiple feeders.

Trisacryl gelatin microspheres have been demonstrated to achieve more distal penetration and higher subsequent devascularization than similarly-sized polyvinyl alcohol [7]. In our series, in one patient (Patient 2), for whom only TAE of the AChA was performed using microspheres, 97.9 % devascularization was achieved. Likewise, in another patient (Patient 3), for whom microsurgical occlusion of the AChA was performed, 98.6 % devascularization was achieved and the tumor was softened. Microsurgical occlusion of feeders alone  also helped to devascularize the tumor, to reduce intraoperative blood loss, and to soften the tumor. Therefore, microsurgical occlusion alone could be an effective and alternative method for feeder obliteration when catheterization of the AChA is difficult.

### Approach used for tumor removal and surgical outcomes

The optimal surgical approach for treating tumors in the lateral ventricle remains controversial. The main reported approaches have been described [1–5, 9, 28]. (1) The trans-parietal approach through the superior parietal lobe or intraparietal sulcus is preferably used, since this approach can reduce the risk of injury to the optic radiation, but can be disadvantageous, depending on the tumor location, due to the long distance to the trigone of the lateral ventricle. In this study, in two patients (Patients 5 and 6) in whom the tumor was firm, we abandoned this approach in favor of the wide parieto-occipital corticectomy in a subsequent session. (2) The trans-temporal approach (including the temporo-occipital approach) involves a route through the middle or inferior temporal gyrus. This route provides for a shorter distance to reach the tumor, but may increase the risk of injury to the inferior fibers of the optic radiation and to the language cortices in the dominant hemisphere. We used this approach in one patient (Patient 4) after receiving consent since the patient already had homonymous hemianopia prior to the surgery; the symptom had not resolved by the time of the last-follow up due to injury to the optic radiation. (3) The interhemispheric parieto-occipital precuneus approach involves a route associated with a low incidence of hemianopia, aphasia, and epilepsy. We did not select this approach in any of the six patients  since a wide brain retraction was necessary, and  due to  risks of injury or thrombosis of bridging veins or the sagittal sinus.

In a recent review, the postoperative mortality rate following treatment of intraventricular meningiomas was 4.0 %; most deaths were related to hematomas in the surgical bed, probably due to rich tumor vascularization [6]. In this study, the mortality was 0 % and mRS scores assessed at the last-follow up were ≤ 2 in all patients. Notably, no postoperative hemorrhage had occured in this study, probably due to feeder obliteration prior to the tumor removal; therefore, although the staged treatment was required, preceding feeder obliteration could limit intraoperative blood loss and prevent postoperative hemorrhages, and the treatment outcomes of these patients were relatively favorable.

### Limitations

This study has several limitations. First, as large intraventricular tumors are relatively rare, the number of cases we assessed was too small to confirm the genuine efficacy of feeder obliteration preceding the surgical tumor removal. Second, the present study was not randomized, which makes outcome comparisons difficult. However, compared with the outcomes of current treatment options for patients with these types of tumors described in the literature [1, 6], the outcomes observed in this study were relatively favorable. Notably, in this study, no patients had permanent neurological deficits due to feeder obliteration. Further multicenter, prospective studies are needed to verify the efficacy of feeder obliteration preceding direct surgery and/or endovascular treatment of these tumors.

## Conclusions

We have described the results of persistent feeder obliteration of choroidal arteries by endovascular treatment and/or microsurgery prior to surgical removal of large hypervascular tumors in the lateral ventricle. In our study, the outcomes were relatively favorable, but further case accumulation is warranted to confirm the clinical efficacy and safety of this procedure.

## Data Availability

The datasets used and/or analyzed during the current study are available from the corresponding author on reasonable request.
